# A Rare Case of Splenic Metastasis From Squamous Cell Carcinoma of the Cervix Detected on 18F-Fluorodeoxyglucose PET/CT

**DOI:** 10.7759/cureus.31974

**Published:** 2022-11-28

**Authors:** Abhishek Kumar, Amitabh Upadhyay, Vanita Pandey, Bhola Kumar, Sujata Mitra

**Affiliations:** 1 Nuclear Medicine, Tata Main Hospital, Jamshedpur, IND; 2 Oncology, Tata Main Hospital, Jamshedpur, IND; 3 Pathology, Meherbai Tata Memorial Hospital, Jamshedpur, IND

**Keywords:** 18f-fluorodeoxyglucose, positron emission tomography, 18f-fluorodeoxyglucose-positron emission tomography, cervical cancer, splenic metastasis, carcinoma cervix, squamous cell carcinoma, pet/ct, 18f - fdg

## Abstract

Cervical cancer is one of the common gynaecological malignancies seen in women. As per the World Health Organization (WHO), cervical cancer is the fourth most common malignancy encountered in women worldwide. Squamous cell carcinoma followed by adenocarcinoma is the most common histological subtype of cervical cancer. Apart from nodal metastases, the usual sites of metastases are the lungs, bones, and liver. Spleen, breast, and skin have been reported as rare sites of metastasis in cases of cervical cancer. Spleen is a rare site of metastasis not only in cases of carcinoma cervix but also in various other solid tumour malignancies. Splenic metastases being uncommon are difficult to characterise using routine imaging modalities. 18F-fluorodeoxyglucose positron emission tomography/computed tomography (18F-FDG PET/CT) evaluation helps to detect these rare sites of metastasis.

## Introduction

18F-fluorodeoxyglucose (18F-FDG) positron emission tomography/computed tomography (PET/CT) imaging is an excellent imaging modality in staging, response evaluation, and detection of recurrences in solid tumour malignancies. 18F-FDG PET combined with contrast-enhanced computed tomography (CECT) imaging is increasingly becoming an initial imaging modality of choice in the evaluation of malignant diseases. 18F-FDG localises to cells with increased glucose metabolism in the body and also in the tumour cells, which also have increased glucose metabolism. This accumulation of 18F-FDG in tumour cells helps in the localisation and assessment of malignant primary disease as well as metastatic sites. In cases of cervical cancer, 18F-FDG PET/CT is used to detect metastasis in regional lymph nodes and distant sites. It also supplements magnetic resonance imaging (MRI) in the evaluation of primary neoplastic disease. It is increasingly being used for planning imaging-guided brachytherapy and helps reduce the radiation burden in normal soft tissue by accurately defining the primary disease and metastatic sites [1]. The usual sites of metastasis in cervical cancer are regional lymph nodes followed by lungs, bones, and liver [2-4]. Rare sites of metastasis are the spleen, breast, and skin [5-7]. We report a case of splenic metastasis in squamous cell carcinoma of the cervix detected on 18F-FDG PET/CT.

## Case presentation

**Figure 1:**
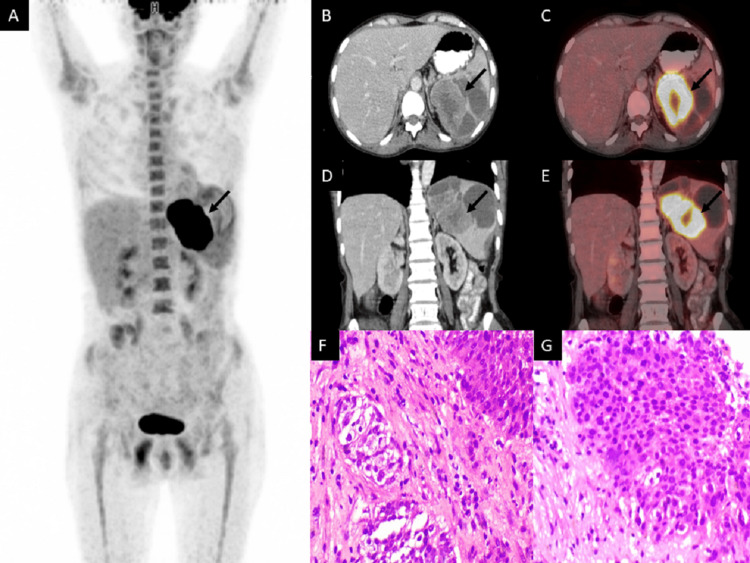
18F-FDG PET/CT and histopathology images of the patient (A) MIP; (B) axial view of CT; (C) axial view of 18F-FDG PET/CT fusion; (D) coronal view of CT; (E) coronal view of 18F-FDG PET/CT fusion; (F and G) H&E staining histopathology images at 40x magnification. MIP images show increased FDG uptake in the region of the spleen (arrow). Axial and coronal CT and 18F-FDG PET/CT fusion images reveal cystic lesions in the spleen with soft tissue components showing FDG uptake (arrow). Histopathology images reveal infiltrating discrete and confluent lobules of neoplastic squamoid cells, with no evidence of keratinisation, but visible intercellular bridges, against a desmoplastic background with infiltrates of lymphoid cells. High mitotic activity and lymphovascular emboli are also noted. MIP: maximal intensity projection; 18F-FDG PET/CT: 18F-fluorodeoxyglucose positron emission tomography/computed tomography; FDG: fluorodeoxyglucose; H&E: hematoxylin and eosin.

The patient was a 46-year-old woman who underwent a hysterectomy for dysfunctional uterine bleeding. Histopathology confirmed the diagnosis as squamous cell carcinoma of the cervix. CT of the chest and abdomen were done for metastatic workup. She underwent chemoradiation as further treatment. After seven months of completing chemoradiation, she presented with small bowel obstruction. Exploratory laparotomy was undertaken with a diversion loop ileostomy. Intra-operatively, peritoneal deposits were noted. Further evaluation with CT of the abdomen revealed recurrent metastatic disease in the form of pelvic mass and peritoneal deposits. Hypodense splenic lesions were also noted, which could not be characterized on CT. In view of extensive disease, the patient was put on systemic palliative chemotherapy. Follow-up response evaluation CT after chemotherapy revealed resolution of pelvic mass and peritoneal deposits; however, persistent splenic lesions were still noted. Subsequently, 18F-FDG PET/CT was advised for evaluation of splenic lesions and restaging of the disease. 18F-FDG PET/CT revealed cystic lesions in the spleen with associated fluorodeoxyglucose (FDG)-avid enhancing soft tissue component (Figure 1). No other site of abnormal FDG uptake was noted in the rest of the PET CT to suggest metastatic disease. These findings further raised the suspicion of splenic metastasis. The patient was reluctant to undergo splenectomy as further treatment. Biopsy from the splenic lesion was then undertaken and histopathology revealed infiltrating discrete and confluent lobules of neoplastic squamoid cells, with no evidence of keratinisation, but visible intercellular bridges noted against a desmoplastic background with infiltrates of lymphoid cells. High mitotic activity and lymphovascular emboli were noted, with a histological opinion of metastatic carcinoma. Upon immunohistochemistry, cytokeratin, p40 markers of squamous differentiation, and p16 as diffuse and strong nuclear and cytoplasmic expression were noted, and the case was opined as metastatic non-keratinising squamous cell carcinoma, human papillomavirus (HPV) associated, from known primary cervical cancer. The patient was further continued with systemic palliative chemotherapy.

## Discussion

As per the WHO, cervical cancer is the fourth most common malignancy encountered in women worldwide. Cervical cancer is prevalent cancer and a leading cause of death among females in low and middle-income countries. Cervical cancer commonly presents with postcoital bleeding, vaginal discharge, or pelvic pain. Local examination and cervical biopsy are used for confirmation of diagnosis. Like any other malignancy, early detection and treatment of cervical cancer lead to better survival outcomes. Routine screening with Papanicolaou (PAP) smear cytology and detection of precancerous lesions is now recommended for women. Screening can begin at the early age of 21 years. Cervical cancer is frequently associated with HPV infection, which is a sexually transmitted disease. The WHO now encourages using HPV tests for cervical screening as well, including HPV DNA and HPV mRNA tests. The introduction of HPV immunization is a novel and promising approach to the prevention of cervical cancer [8]. Bivalent, quadrivalent, and nonavalent vaccines are available to protect against infection with various HPV subtypes. These vaccines protect against infection with various HPV subtypes, particularly HPV 16 and 18, which cause the majority of cervical cancers and precancers. These vaccines are recommended for young adolescents and teenage boys and girls. Some countries also recommend HPV vaccination for adults. This vaccination, however, does not eliminate the need for screening later in life as it does not protect against infection from all the variants of HPV. In low and middle-income countries, raising cancer awareness in the general population remains an invaluable tool in the early detection of cervical cancer, particularly when HPV immunisation is not accessible for all.

Once the diagnosis of cervical cancer is established, staging is done as per the International Federation of Gynecology and Obstetrics (FIGO) classification. MRI is the imaging modality of choice in the evaluation of primary lesions in cervical cancer. It helps stratify patients for surgery and radiation therapy. Determination of pretreatment nodal metastasis is an important prognostic factor in the progression-free survival of patients with cervical cancer. 18F-FDG PET/CT and MRI are now routinely used for the detection of nodal metastasis on imaging [9]. The usual metastatic sites in carcinoma cervix are pelvic lymph nodes followed by paraaortic nodes and then distant metastasis. The usual distant metastatic sites are the lungs, bone, liver, and peritoneum. Rare sites of metastasis such as muscles, bowel loops, spleen, breast, and skin have been reported in the literature [5-7]. After the staging of the disease, these patients are offered treatment modalities in the form of surgery, radiotherapy, and concurrent chemoradiation depending on the stage. Surgery is the standard of treatment in early-stage cervical cancer [10,11]. Surgical treatments include fertility-sparing conization, simple hysterectomy, or hysterectomy with full pelvic lymphadenectomy. Radiotherapy plays a critical role in the management of cervical cancer. It is used as an adjuvant treatment for patients with high-risk pathologic features. It also has a definitive role in locoregionally advanced disease [12]. Concurrent chemotherapy with either cisplatin and/or 5‐fluorouracil has been used in the treatment of locally recurrent disease [13]. Cisplatin and paclitaxel-based systemic palliative chemotherapy are used in patients with distant metastatic disease. Patients with extensive local disease or distant metastatic disease are assigned to palliative therapy with supportive care [14]. 18F-FDG PET/CT and MRI are also used in suspected recurrent cervical cancer after surgery or radiotherapy. Stojiljkovic et al. compared MRI and 18F-FDG PET/CT in the detection of recurrent cervical cancer after radiotherapy. They reported sensitivity, specificity, and accuracy of MRI vs. 18F-FDG PET/CT as 80.1%, 52.4%, and 66.7% vs. 97.6%, 61.9%, and 79.8%, respectively [15]. Splenic metastases in cervical cancer are rare and have been reported in published literature. Aitelhaj et al. and Taga et al. have earlier reported splenic metastasis in cases of cervical cancer [5,6]. In the present case report, 18F-FDG PET/CT guided the site of biopsy, which was done from the part of the lesion with FDG uptake. Thus emphasizing the role of 18F-FDG PET/CT in the guidance of biopsy sites.

## Conclusions

The current case highlights the importance of 18F-FDG PET/CT imaging in patients with cervical cancer. It is useful in the initial evaluation and staging of cervical cancer as well as in response evaluation and detection of recurrent disease. It has a role in radiation treatment planning for targeted therapy and reduces radiation burden in adjacent structures. With the introduction of PET/MRI imaging, there is an advantage of combining the benefits of 18F-FDG PET and MRI together in one single modality and it might as well be the future of oncology imaging.
